# Suspected azathioprine induced liver cirrhosis: an unusual side effect

**DOI:** 10.11604/pamj.2014.17.174.3018

**Published:** 2014-03-07

**Authors:** Aida Ben Slama Trabelsi, Eya Hamami, Ahlem Souguir, Mehdi Ksiaa, Salem Ajmi, Ali Jmaa

**Affiliations:** 1 Department of Gastroenterology, Sahloul Sousse, Tunisia

**Keywords:** Azathioprine, Crohn′s disease, hepatotoxicity, liver cirrhosis

## Abstract

In recent years, the hepatotoxic potential of thiopurines, in particular 6-thioguanine (6-TG) has been discussed in literature. However, cirrhosis was exceptionally reported. We report the case of a 56-year-old woman with ileocaecal Crohn's disease treated with azathioprine. After taking azathioprine (2 mg/kg daily) for four years, she underwent surgical treatment for acute intestinal obstruction. In peroperative, we noticed a cirrhotic liver. A surgical biopsy was performed and the diagnosis of cirrhosis was confirmed. Autoimmune and viral liver diseases were ruled out by laboratory parameters. Therefore, Azathioprine is believed to be the causative actor for inducing liver cirrhosis. Thus, treating inflammatory bowel disease effectively while trying to limit iatrogenic disease is a continuous struggle.

## Introduction

Nowadays, thiopurines (azathioprine (AZA) and mercaptopurine (6 MP)) are the most commonly used immunomodulatory drugs for managing patients with inflammatory bowel disease (IBD)[1]. They are among the pharmacological agents with the greatest potential to cause adverse reactions. The side-effects of thiopurines can be divided into dose independent or “allergic/idiosyncratic” and dose-dependent events. Hepatic toxicity is believed to be a dose independent side effect of AZA. In recent years the hepatotoxic profile of thiopurines has been recognised. Most hepatic lesions described are vascular, such as peliosis hepatis, veno-occlusive disease, perisinusoidal fibrosis, hepatoportal sclerosis, and nodular regenerative hyperplasia. Even after long term treatment, most series report a rate of hepatic abnormalities of between 3 and 10%, which are usually limited to abnormal liver function tests and minor changes seen on liver biopsy specimens. The occurrence of side-effects, however, is a major drawback in the use of AZA or MP[1]. Cirrhosis is an exceptional complication of thiopurine drugs; only one case was reported in literature [2]. We report a rare case of liver cirrhosis in a Crohn′s disease patient associated with AZA therapy and we try to do a review of the literature regarding this complication.

## Patient and observation

A 56 year old woman was followed, for 20 years, for ileocaecal Crohn disease complicated with stenosis. A corticosteroid pulse therapy was administered to induce remission. In 2000, a surgical treatment consisting of an ileocaecal resection was performed due to an acute intestinal obstruction caused by the terminal ileum stenosis. During surgery, liver was macroscopically normal. In the post-operative, Budesonide therapy was immediately initiated. The disease remained quiescent and regular laboratory controls including serum transaminases were normal. In 2006, the patient was treated with AZA in doses of 2mg/kg/j. She remained free of endoscopic and clinical recurrence until 2010 when she underwent reoperation for adhesions bowel obstruction. She had a new resection of the ileum. At laparotomy, the liver was macroscopically fibrosed. A surgical hepatic biopsy specimen was taken for histological examination and confirmed liver cirrhosis. Liver function tests were normal. Viral liver diseases were ruled out by laboratory parameters. Autoimmune diseases of the liver are also unlikely in the absence of autoantibodies. Then, AZA therapy (4 years) was believed to be the causative factor of cirrhosis.

## Discussion

The thiopurine drugs such as AZA and 6-MP represent an effective and widely used immunosuppressant in the therapeutic armamentarium of IBD. They can induce and maintain remission of Crohn's disease (CD) and ulcerative colitis (UC), and have steroid-sparing effects in patients with steroid-dependent IBD [3, 4]. However, their therapeutic role is disputable because of toxicity. Up to 25% of patients may be unable to continue the drug due to side effects. The incidence of hepatotoxicity associated with thiopurine use is reported between 0% and 32% [5]. Many of the symptoms of hepatotoxicity can be nonspecific and can be confused with a flare-up of inflammatory bowel disease. As well, the subtype resulting in portal hypertension can occur without biochemical abnormalities. Thiopurine-induced hepatotoxicity can be grouped into three syndromes: hypersensitivity, idiosyncratic cholestatic reaction, and endothelial cell injury (with resultant raised portal pressures, veno-occlusive disease, or peliosis hepatis, perisinusoidal fibrosis and nodular regenerative hyperplasia) [6].

AZA and 6-MP are metabolized into active and inactive metabolites by the same enzymatic cascade. AZA is a pro-drug that is converted to 6-MP via a nonenzymatic metabolic pathway and its imidazole derivative by glutathione in the liver. Then, 6-MP enters cells and is subject to 3 competing enzymatic pathways [7]. It may be activated via a multi-step enzymatic pathway to produce the active metabolites, the 6-thioguanine nucleotides (6-TGNs). 6-MP is also metabolized bythiopurine methyl transferase (TPMT) to 6-methylmercaptopurine (6-MMP) or by xanthine oxidase to 6-thiouric acid [7]. Several metabolites have been held responsible for induction of adverse events. Many studies have shown that hepatotoxicity seems to be related to the accumulation of methylated metabolites such as 6-MMP [2, 8]. The enzyme TPMT is the key enzyme in the metabolic pathway: patients with very high TPMT activity are resistant to thiopurine drugs due to shunting of 6-MP away from 6 TGN towards over production of 6-MMP [8, 9], and at the risk of hepatotoxicity due to high 6-MMP concentrations[10].

In our case, high TPMT activity cannot be the mechanism of hepatotoxicity. Indeed, azathioprine was effective in maintaining remission in our patient at a dose of 2mg / kg / day. The hypothesis that high 6-TGN levels are hepatotoxic may provide an explanation why our patient developed liver cirrhosis. The higher occurrence of histological liver abnormalities during 6-TG treatment in comparison with AZA or 6-mercaptopurine (6-MP) may be explained by the significantly higher levels of 6-TGN reached by 6-TG [2].

## Conclusion

This case illustrates the potential toxicity of AZA, highlights the need to monitor liver function tests in patients treated with thiopurine, and identifies the need for additional research focused on the mechanism of thiopurine-induced hepatic injury in patients treated with thiopurines for inflammatory bowel disease. Cirrhosis may be an exceptional complication of thiopurine drugs. Knowledge of such side effect could justify the routine use of abdominal ultrasound in monitoring patients on thiopurine as liver function tests may be normal.
